# Editorial: Autophagy: From Big Data to Physiological Significance

**DOI:** 10.3389/fcell.2019.00376

**Published:** 2020-01-10

**Authors:** Sovan Sarkar, Maria I. Vaccaro, Ioannis P. Nezis

**Affiliations:** ^1^College of Medical and Dental Sciences, Institute of Cancer and Genomic Sciences, Institute of Biomedical Research, University of Birmingham, Birmingham, United Kingdom; ^2^Department of Pathophysiology, School of Pharmacy and Biochemistry, Institute of Biochemistry and Molecular Medicine, University of Buenos Aires, National Council for Scientific Research (CONICET), Buenos Aires, Argentina; ^3^School of Life Sciences, University of Warwick, Coventry, United Kingdom

**Keywords:** autophagy, xenophagy, data, disease, screening

Autophagy is a fundamental catabolic process where cytoplasmic components are sequestered into double-membrane vesicles called autophagosomes, which then fuse with lysosomes and their content is degraded. Our knowledge about autophagy sharply increased during the last decade. This significant progress helped us to understand better the molecular mechanisms of autophagy and to elucidate its role in health and disease. This special issue contains a collection of three original research papers and 12 review articles covering a broad range of topics highlighting how big data and screening approaches can help toward uncovering the molecular mechanisms of autophagy.

Recent years have witnessed the development of large-scale multi-omics studies on autophagy via genomics, transcriptomics, proteomics, lipidomics, and metabolomics. Jacomin et al. comprehensively describe the omics studies undertaken in the field of autophagy, and the integration of these omics datasets for better understanding of autophagy regulation and the involvement of autophagy in other biological processes. In addition, future approaches involving single-cell analysis, patient-derived samples, and high-content analysis have been suggested. The authors also outlined the web-based resources for studying autophagy, such as for the prediction of Atg8-family interacting proteins, and autophagy network and databases. Overall, the emerging big data and *in silico* tools not only elucidate the global landscape of autophagy but also provide critical resources for further research in this field.

There is a growing interest toward the biomedical exploitation of autophagy modulators for the treatment of myriad human diseases. Two articles comprehensively review the screening methods for the drug discovery of chemical autophagy modulators. The first article by Panda et al. summarizes the *in vitro* chemical screening approaches for identifying autophagy modulators in mammalian cells. These methods that are commonly being used, involve reporters based on the autophagic marker LC3 or specific autophagy substrates like p62 and certain aggregation-prone proteins. The chemical screenings pertaining to the discovery of the pharmacological modulators of autophagy have been described. Of biomedical relevance, the therapeutic benefits of autophagy modulators have been highlighted in animal and iPSC models of selected human diseases, such as in neurodegenerative disorders, cancer, infectious diseases, liver diseases, and myopathies, as well as in lifespan extension. The second article by Mishra et al. primarily focuses on the chemical biology strategies utilizing high-throughput assays to monitor autophagy in yeast and mammalian cells. These assays are based on the growth of yeast cells, fluorescence readouts of LC3 reporters in mammalian cells, and luminescence measurements of autophagic cargo clearance including organelle turnover in both yeast and mammalian cells. Apart from describing the therapeutic applications of autophagy modulators, how these compounds act as valuable tools to elucidate the regulation of autophagy have also been highlighted.

For developing novel autophagy modulators, high-throughput screens were undertaken in the research article by Pengo et al. for identifying the regulators of ATG4B activity. The protease ATG4B is a key regulator of the LC3/GABARAP conjugation system essential for autophagosome formation. Inhibition of ATG4B activity has been suggested for cancer treatment. Through chemical and genetic screens utilizing a cellular luciferase-based assay for measuring ATG4B activity, the compound STK683963 and the kinase AKT2 were identified as activators. Although this study focused on the enhancers of ATG4B activity, these regulators could impact on the kinetics of LC3/GABARAP processing and influence autophagy. The datasets of ATG4B modulators arising from the screens have been provided for further investigation.

There is significant development in the understanding of the molecular mechanisms of autophagy regulation, such as the initial steps of autophagosome biogenesis in mammals. The review article by Grasso et al. provides a detailed overview of the early events in mammalian autophagosome formation including their membrane origins and cellular localization. The four major aspects outlined in this article encompass autophagy induction via physiological stressors, autophagy initiation via mTOR and AMPK, initiation of autophagosome formation via the ULK1 complex, and the molecular mechanisms of phagophore generation prior to autophagosome formation.

Although it was initially believed to be a bulk process, it is now well-established that autophagy is a selective process. Xenophagy is a type of selective autophagy and refers to the selective autophagic degradation of invading bacteria and viruses, and is an important aspect of the hosts' innate immune response to protect against infection. Three review articles in this collection highlight the importance of xenophagy in diseases. Depending on the virus, autophagy can restrict or promote viral replication, and play key roles in modulating inflammation and cell survival. Ahmad et al. provide an overview of autophagy-virus interplay highlighting the protective role of autophagy in human infections. They summarize recent discoveries showing the role of autophagy in immunity and inflammation upon viral infection. Finally, they discuss therapeutic implications and potential caveats associated with using autophagy to control viral infections in humans. Sharma et al., focus on bacterial degradation by autophagy. They describe how several bacterial effectors regulate host autophagy during infection and how this affects inflammation. They also present a detailed overview on the role of several selective autophagy receptors and adaptors on bacterial xenophagy. Finally, they describe how ubiquitin ligases and deubiquitinases regulate bacterial xenophagy. Evans et al., provide a comprehensive overview of the interplay between host autophagy and eukaryotic pathogens. They focus on eukaryotic pathogens *Plasmodium, Toxoplasma, Leishmania*, and the fungal pathogens *Candida albicans, Aspergillus fumigatus*, and *Cryptococcus neoformans*.

Neutrophils are effector cells of immune system in humans and are the first cells to respond to tissue inflammation. Skendros et al. review the role of autophagy in the biology of neutrophils. They describe the link between autophagy and regulation of granulopoiesis and neutrophil degranulation. They also describe how autophagy affects net formation, the extracellular chromatin strands carrying various highly active neutrophil-derived granular and cytosolic proteins. Finally, they explore how elements of autophagic machinery could be effective therapeutic targets for the enhancement of antimicrobial defense or the amelioration of neutrophil/NET-driven inflammation and thrombosis.

Ianniciello et al. explore the relationship between autophagy and metabolism in the leukemic stem cells (LSCs). They give an overview of the metabolic features involved in hematopoietic stem cells (HSCs) that utilize glycolysis and fatty acid oxidation as sources of energy. HSCs develop high levels of autophagy; ATG7, ATG5, and the ULK1 complex have been linked with mitophagy in HSCs. Autophagy, which contributes to fuel LSCs energy demand and hypoxic environment, along with mutations and epigenetics modifications driving LSCs expansion, are proposed to be principal contributors in HSCs leukemic transformation. In conclusion, authors highlight the relevance of combining current treatment with the autophagy inhibitor chloroquine in LSCs.

Di Malta et al. focus in the transcriptional regulation of autophagy, particularly on the role of the MiT/TFEB transcription factor family. TFEB activation not only promotes the increment of lysosomal catabolic efficiency but also controls the expression of ATG genes driving the autophagic flux. The description of the opposed role of ZKSCAN3 and TFEB let us understand the nuclear events that control autophagy. Cytosolic TFEB and nuclear ZKSCAN3 inhibit lysosome gene expression under nutrient starvation conditions. Normoxia and hypoxia conditions also regulate ATG genes such as Bnip3 through NFKB and E2F1. Finally, they propose that the modulation of the transcriptional control of autophagy could be considered as possible therapeutic strategies for complex diseases.

Kocaturk and Gozuacik describe the relationship between autophagy and ubiquitin proteasome system (UPS). Both degradative mechanisms are linked by the ubiquitin signaling pathway. Proteins with K48-based ubiquitin chains are directed for UPS and aggregates with K63-based ubiquitin chains are directed for autophagic degradation. Both K48- and K63-linked ubiquitylation were observed in cases of xenophagy, which is an example of coregulation of the UPS and autophagy. In addition, UPS and autophagy act as cooperative mechanisms in mitophagy, peroxiphagy, and ERphagy. Moreover, UPS can regulate degradation of transcription factors involved in autophagy. Eventually, this article discusses the possible role of the cross talking between autophagy and UPS in degenerative diseases and cancer.

Daskalaki et al. present a comprehensive summary of recent findings on selective autophagy in hypoxia and discuss emerging links between these pathways and cancer pathophysiology. In response to hypoxia, HIF-1 is stabilized and translocate to the nucleus to initiate the transcription of multiple genes involved in autophagy, glucose metabolism and mitochondria respiration. Importantly, HIF-1 regulates essential genes for the assembly and function of the autophagy machinery. This article also focuses in the role of FUN14 domain-containing protein 1(FUNDC1) in the regulation of mitophagy in normoxia vs. hypoxia. Furthermore, hypoxia induces degradation of other organelles by selective autophagy and the components of these selective pathways in cancer are discussed.

In the research article by, Pérez et al. a role of lysosome-associated membrane protein LAMP-2C in the regulation of melanoma growth and survival is presented. They show that melanoma cell expression of LAMP2C mRNA significantly increased in response to pro-inflammatory cytokine interferon-gamma. This increased expression affected macroautophagy and chaperone-mediated autophagy in several human melanoma lines. Melanoma cells with enhanced LAMP-2C expression displayed increased cell cycle arrest, increased expression of Chk1 and p21, and greater apoptosis and necrosis. In addition, human melanoma cell xenografts with increased LAMP-2C expression, displayed reduced growth in immune compromised murine hosts. Melanomas with high LAMP-2C expression showed increased necrosis and reduced cell density upon histological analysis.

Nilangekar et al., developed new genetic tools to study autophagy in the context of gametogenesis and germline stem cell aging. They generated three transgenic lines mCherry-Atg8a, GFP-Ref(2)P, and mito-roGFP2-Orp1 that are specifically expressed in the germline compartment during Drosophila oogenesis. These reporters can be used to monitor and quantify autophagy and the production of reactive oxygen species during oogenesis. They are a valuable tool that can be used in designing genetic screens to identify novel regulators of autophagy and redox homeostasis during oogenesis.

## Author Contributions

All authors listed have made a substantial, direct and intellectual contribution to the work, and approved it for publication.

### Conflict of Interest

The authors declare that the research was conducted in the absence of any commercial or financial relationships that could be construed as a potential conflict of interest.

